# Supplementary material for: A systematic review of clinical health conditions predicted by machine learning diagnostic and prognostic models trained or validated using real-world primary health care data
Source: PLoS One. 2023 Sep 8;18(9):e0274276. doi: 10.1371/journal.pone.0274276 (PMC10491005; doi:10.1371/journal.pone.0274276)
Supplement: S1 Table — (PDF) [file pone.0274276.s001.pdf]

S1 Table. List of excluded studies with reasons (n=58): Not PHC data (n=34), Unclear data source (n=11), Not ML technique (n=5), Not ML prediction model (n=4), Not real-world data (n=3), No targeted health conditions (n=1).

Reference	Reason of Exclusion
Adal KM, Sidibé D, Ali S, Chaum E, Karnowski TP, Mériaudeau F. Automated detection of microaneurysms using scale-adapted blob analysis and semi-supervised learning. Comput Methods Programs Biomed. 2014;114: 1–10. doi:10.1016/J.CMPB.2013.12.009	Not PHC data
Akella A, Kaushik V. Machine Learning Algorithms for Predicting Coronary Artery Disease: Efforts Toward an Open Source Solution. bioRxiv. 2020; 2020.02.13.948414.	Not PHC data
Alaa AM, Bolton T, Di Angelantonio E, Rudd JH, Van Der Schaar M. Cardiovascular disease risk prediction using machine learning: A prospective cohort study of 423,604 participants. Circulation. 2018;138.	Not PHC data
Althnian A, Elwafa AA, Aloboud N, Alrasheed H, Kurdi H. Prediction of COVID-19 Individual Susceptibility using Demographic Data: A Case Study on Saudi Arabia. Procedia Comput Sci. 2020;177: 379–386.	Unclear data source
Andrisevic N, Ejaz K, Rios-Gutierrez F, Alba-Flores R, Nordehn G, Burns S. Detection of heart murmurs using wavelet analysis and artificial neural networks. J Biomech Eng. 2005;127: 899–904. doi:10.1115/1.2049327	Not PHC data
Basu S, Raghavan S, Wexler DJ, Berkowitz SA. Characteristics Associated With Decreased or Increased Mortality Risk From Glycemic Therapy Among Patients With Type 2 Diabetes and High Cardiovascular Risk: Machine Learning Analysis of the ACCORD Trial. Diabetes Care. 41: 604–612.	Unclear data source
Baxter SL, Saseendrakumar BR, Paul P, Kim J, Bonomi L, Kuo T-T, et al. Predictive Analytics for Glaucoma Using Data From the All of Us Research Program. Am J Ophthalmol. 2021;227: 74–86.	Not PHC data
Bertoncelli CM, Altamura P, Bertoncelli D, Rampal V, Vieira ER, Solla F. PredictMed: A Machine Learning Model for Identifying Risk Factors of Neuromuscular Hip Dysplasia: A Multicenter Descriptive Study. Neuropediatrics. 2020.	Not PHC data

Bertsias A, Symvoulakis E, Tziraki C, Panagiotakis S, Mathioudakis L, Zaganas I, et al. Cognitive Impairment and Dementia in Primary Care: Current Knowledge and Future Directions Based on Findings From a Large Cross-Sectional Study in Crete, Greece. Front Med. 2020;7: 592924. doi:10.3389/fmed.2020.592924	Not real-world data
Bora A, Balasubramanian S, Babenko B, Virmani S, Venugopalan S, Mitani A, et al. Predicting the risk of developing diabetic retinopathy using deep learning. Lancet Digit Heal. 2020;3: E10–E19.	Not real-world data
Chang C-Y, Lu Y-CA, Ting W-C, Shen T-WD, Peng W-C. An artificial immune system with bootstrap sampling for the diagnosis of recurrent endometrial cancers. Open Med. 2021;16: 237–245.	Not PHC data
Dascalu A, David EO. Skin cancer detection by deep learning and sound analysis algorithms: A prospective clinical study of an elementary dermoscope. EBioMedicine. 2019;43: 107–113.	Not PHC data
de Cos Juez FJ, Suarez-Suarez MA, Sanchez Lasheras F, Murcia-Mazon A. Application of neural networks to the study of the influence of diet and lifestyle on the value of bone mineral density in post-menopausal women. Math Comput Model. 54: 1665–1670.	Not PHC data
Deodhar A, Rozycki M, Garges C, Shukla O, Arndt T, Grabowsky T, et al. Use of machine learning techniques in the development and refinement of a predictive model for early diagnosis of ankylosing spondylitis. Clin Rheumatol. 39: 975–982.	Unclear data source
Dong J, Buas MF, Gharahkhani P, Kendall BJ, Onstad L, Zhao S, et al. Determining Risk of Barrett's Esophagus and Esophageal Adenocarcinoma Based on Epidemiologic Factors and Genetic Variants. Gastroenterology. 2018;154: 1273-1281.e3.	Not PHC data
Fu YK, Liu HM, Lee LH, Chen YJ, Chien SH, Lin JS, et al. The TVGH-NYCU Thal-Classifier: Development of a Machine-Learning Classifier for Differentiating Thalassemia and Non-Thalassemia Patients. Diagnostics (Basel). 2021/09/29. 2021;11. doi:10.3390/diagnostics11091725	Not PHC data
Funston G, Abel G, Crosbie EJ, Hamilton W, Walter FM. Could ovarian cancer prediction models improve the triage of symptomatic women in primary care? A modelling study using routinely collected data. Cancers (Basel). 2021;13.	Not ML technique
Giavina-Bianchi M, de Sousa RM, Paciello VZA, Vitor WG, Okita AL, Proa R, et al. Implementation of artificial intelligence algorithms for melanoma screening in a primary care setting. PLoS One. 2021/09/23. 2021;16: e0257006. doi:10.1371/journal.pone.0257006	Not PHC data

Goto S, Goto S, Pieper KS, Bassand JP, Camm AJ, Fitzmaurice DA, et al. New artificial intelligence prediction model using serial prothrombin time international normalized ratio measurements in atrial fibrillation patients on vitamin K antagonists: GARFIELD-AF. Eur Hear J Cardiovasc Pharmacother. 2019/12/11. 2020;6: 301–309. doi:10.1093/ehjcvp/pvz076	Unclear data source
Grill E, Groezinger M, Feil K, Strupp M, Hoerbst A, Hackl WO, et al. Developing and Implementing Diagnostic Prediction Models for Vestibular Diseases in Primary Care. 2016. pp. 735–739.	Not PHC data
Horowitz N, Moshkowitz M, Halpern Z, Leshno M. Applying data mining techniques in the development of a diagnostics questionnaire for GERD. Dig Dis Sci. 2007;52: 1871–1878. doi:10.1007/s10620-006-9202-5	Not PHC data
Jonnagaddala J, Liaw S-T, Ray P, Kumar M, Dai H-J, Hsu C-Y. Identification and Progression of Heart Disease Risk Factors in Diabetic Patients from Longitudinal Electronic Health Records. BioMed Res Int. 2015;2015.	Unclear data source
Jordan P, Shedden-Mora MC, Löwe B. Predicting suicidal ideation in primary care: An approach to identify easily assessable key variables. Gen Hosp Psychiatry. 2018;51: 106–111. doi:10.1016/j.genhosppsych.2018.02.002	Not real-world data
Kaneko H, Umakoshi H, Ogata M, Wada N, Iwahashi N, Fukumoto T, et al. Machine learning based models for prediction of subtype diagnosis of primary aldosteronism using blood test. Sci Rep. 2021;11: 9140. doi:10.1038/s41598-021-88712-8	Not PHC data
Lazuardi L, Sanjaya GY, Candradewi I, Holmner Å. Automatic platelets counter for supporting dengue case detection in primary health care in indonesia. Stud Health Technol Inform. 2013;192: 585–588.	Not ML technique
Li T, Chien Y, Chou C, Liao C, Cheah W, Fu L, et al. A Fast and Low-Cost Repetitive Movement Pattern Indicator for Massive Dementia Screening. 2020;17.	Not PHC data
Li Y-H, Sheu WH-H, Chou C-C, Lin C-H, Cheng Y-S, Wang C-Y, et al. The Clinical Influence after Implementation of Convolutional Neural Network-Based Software for Diabetic Retinopathy Detection in the Primary Care Setting. Life-Basel. 2021;11: 200. doi:10.3390/LIFE11030200	Not PHC data
Lindow T, Kron J, Thulesius H, Ljungström E, Pahlm O. Erroneous computer-based interpretations of atrial fibrillation and atrial flutter in a Swedish primary health care setting. Scand J Prim Health Care. 2019;37: 426–433. doi:10.1080/02813432.2019.1684429	Not ML technique
Mar T, Zaunseder S, Martínez JP, Llamedo M, Poll R. Optimization of ECG Classification by Means of Feature Selection. 2011;58.	Not PHC data

Medina-Inojosa J, Ladejobi A, Attia Z, Shelly-Cohen M, Gersh B, Noseworthy P, et al. The association of artificial intelligence-enabled electrocardiogram-derived age (physiologic age) with atherosclerotic cardiovascular events in the community. Eur Hear J. 2020;41: 2905.	Not ML prediction model
Morejon A, Mayo-Iscar A, Martin R, Ussa F. Development of a new algorithm based on FDT Matrix perimetry and SD-OCT to improve early glaucoma detection in primary care. Clin Ophthalmol. 2019;13: 33–42. doi:10.2147/OPTH.S177581	Not PHC data
Nori VS, Hane CA, Crown WH, Au R, Burke WJ, Sanghavi DM, et al. Machine learning models to predict onset of dementia: A label learning approach. Alzheimers Dement (N Y). 2019/12/28. 2019;5: 918–925. doi:10.1016/j.trci.2019.10.006	Unclear data source
Ohno-Machado L. A comparison of Cox proportional hazards and artificial neural network models for medical prognosis. Comput Biol Med. 1997;27: 55–65.	Unclear data source
Olling K, Nyeng DW, Wee L. Predicting acute odynophagia during lung cancer radiotherapy using observations derived from patient-centred nursing care. Tech Innov patient Support Radiat Oncol. 2018;5: 16–20. doi:10.1016/j.tipsro.2018.01.002	Not PHC data
Passos IC, Mwangi B, Cao B, Hamilton JE, Wu M-J, Zhang XY, et al. Identifying a clinical signature of suicidality among patients with mood disorders: A pilot study using a machine learning approach. J Affect Disord. 2016;193: 109–116.	Not PHC data
Pearce C, McLeod A, Rinehart N, Whyte R, Deveny E, Shearer M. Artificial intelligence and the clinical world: a view from the front line. Med J Aust. 2019;210 Suppl: S38–S40. doi:10.5694/mja2.50025	No targeted health conditions
Pepplinkhuizen S, Ibrahim S, Vink R, Groot B, Stroes ESG, Bax WA, et al. Electronic health records to facilitate continuous detection of familial hypercholesterolemia. Atherosclerosis. 2020;310: 83–87.	Not ML technique
Piniés JA, González-Carril F, Arteagoitia JM, Irigoien I, Altzibar JM, Rodriguez-Murua JL, et al. Development of a prediction model for fatal and non-fatal coronary heart disease and cardiovascular disease in patients with newly diagnosed type 2 diabetes mellitus: the Basque Country Prospective Complications and Mortality Study risk engine (BASCORE). Diabetologia. 2014;57: 2324–2333. doi:10.1007/s00125-014-3370-1	Not ML technique

Requena-Méndez A, Bussion S, Aldasoro E, Jackson Y, Angheben A, Moore D, et al. Cost-effectiveness of Chagas disease screening in Latin American migrants at primary health-care centres in Europe: a Markov model analysis. Lancet Glob Heal. 2017;5: e439–e447.	Not ML prediction model
Rosenfeld A, Graham DG, Jevons S, Ariza J, Hagan D, Wilson A, et al. Development and validation of a risk prediction model to diagnose Barrett's oesophagus (MARK-BE): a case-control machine learning approach. Lancet Digit Heal. 2020;2: E37–E48. doi:10.1016/S2589-7500(19)30216-X	Not PHC data
Savage RS, Messenger M, Neal RD, Ferguson R, Johnston C, Lloyd KL, et al. Development and validation of multivariable machine learning algorithms to predict risk of cancer in symptomatic patients referred urgently from primary care. medRxiv. 2021; 2020.10.23.20218198.	Not PHC data
Shao Y, Zeng QT, Chen KK, Shutes-David A, Thielke SM, Tsuang DW. Detection of probable dementia cases in undiagnosed patients using structured and unstructured electronic health records. BMC Med Inform Decis Mak. 19.	Not PHC data
Shim J-G, Kim DW, Ryu K-H, Cho E-A, Ahn J-H, Kim J-I, et al. Application of machine learning approaches for osteoporosis risk prediction in postmenopausal women. Arch Osteoporos. 2020;15: 169. doi:10.1007/s11657-020-00802-8	Not PHC data
Soriano LC, Zong J, Rodriguez LAG. Feasibility and validity of the thin database to characterize small cell lung cancer. Pharmacoepidemiol Drug Saf. 2017;26: 306.	Not ML prediction model
Tamminen J, Kallonen A, Hoppu S, Kalliomäki J. Machine learning model predicts short-term mortality among prehospital patients: A prospective development study from Finland. Resusc Plus. 2021;5: 100089.	Not PHC data
Tan T-E, Anees A, Chen C, Li S, Xu X, Li Z, et al. Retinal photograph-based deep learning algorithms for myopia and a blockchain platform to facilitate artificial intelligence medical research: a retrospective multicohort study. Lancet Digit Heal. 2021;3: e317–e329.	Not PHC data
Van Vleck TT, Chan L, Coca SG, Craven CK, Do R, Ellis SB, et al. Augmented intelligence with natural language processing applied to electronic health records for identifying patients with non-alcoholic fatty liver disease at risk for disease progression. Int J Med Inform. 2019;129: 334–341.	Not ML prediction model

Wang L, Blackley S V, Blumenthal KG, Yerneni S, Goss FR, Lo Y-C, et al. A dynamic reaction picklist for improving allergy reaction documentation in the electronic health record. J Am Med Informatics Assoc. 2020;27: 917–923.	Not PHC data
Wang S V, Rogers JR, Jin Y, DeiCicchi D, Dejene S, Connors JM, et al. Stepped-wedge randomised trial to evaluate population health intervention designed to increase appropriate anticoagulation in patients with atrial fibrillation. BMJ Qual Saf. 2019;28: 835–842. doi:10.1136/bmjqs-2019-009367	Not PHC data
Wang W, Ye S, Jiang B, Qian L. Risk factor analysis of bone mineral density based on feature selection in type 2 diabetes. Proceedings - 9th IEEE International Conference on Big Knowledge, ICBK 2018. Department of Endocrinology, First Affiliated Hospital of University of Science and Technology of China, Division of Life Sciences and Medicine, University of Science and Technology of China, Hefei, China; 2018. pp. 221–226. doi:10.1109/ICBK.2018.00037	Not PHC data
Yang F, Banerjee T, Narine K, Shah N. Improving pain management in patients with sickle cell disease from physiological measures using machine learning techniques. Smart Heal. 2018;7: 48–59.	Unclear data source
Yang T, Li F, Zhu B, Chen Y, Chen D, Wang C, et al. An exploratory study of the use of the electronic health records of hypertensive patients to support the primary prevention of stroke in shanghai. Risk Manag Healthc Policy. 2020;13: 1781–1789.	Unclear data source
Yu J, Zhou Y, Yang Q, Liu X, Huang L, Yu P, et al. Machine learning models for screening carotid atherosclerosis in asymptomatic adults. Sci Rep. 2021/11/17. 2021;11: 22236. doi:10.1038/s41598-021-01456-3	Not PHC data
Yuen V, Ran A, Shi J, Sham K, Yang D, Chan VTT, et al. Deep-Learning-Based Pre-Diagnosis Assessment Module for Retinal Photographs: A Multicenter Study. Transl Vis Sci Technol. 2021/09/16. 2021;10: 16. doi:10.1167/tvst.10.11.16	Not PHC data
Zhang J, Gajjala S, Agrawal P, Tison GH, Hallock LA, Beussink-Nelson L, et al. Fully Automated Echocardiogram Interpretation in Clinical Practice. Circulation. 2018;138: 1623–1635. doi:10.1161/CIRCULATIONAHA.118.034338	Not PHC data
Zhang L, Mishra S, Zhang T, Zhang Y, Zhang D, Lv Y, et al. Design and Assessment of Convolutional Neural Network Based Methods for Vitiligo Diagnosis. Front Med. 2021/11/05. 2021;8: 754202. doi:10.3389/fmed.2021.754202	Not PHC data
Zheng L, Wang O, Hao S, Ye C, Liu M, Xia M, et al. Development of an early-warning system for high-risk patients for suicide attempt using deep learning and electronic health records. Transl Psychiatry. 2020;10.	Unclear data source

Zheng T, Xie W, Xu L, He X, Zhang Y, You M, et al. A Machine Learning-based Framework to Identify Type 2 Diabetes through Electronic Health Records. bioRxiv. 2017; 78634.	Unclear data source
