# Supplementary material for: A systematic review of clinical health conditions predicted by machine learning diagnostic and prognostic models trained or validated using real-world primary health care data
Source: PLoS One. 2023 Sep 8;18(9):e0274276. doi: 10.1371/journal.pone.0274276 (PMC10491005; doi:10.1371/journal.pone.0274276)
Supplement: S2 Table — (PDF) [file pone.0274276.s002.pdf]

Supplementary Table

S2 Table: Characteristics of the included ML predictive models (Adopted and modified from [141])

	Advantages	Disadvantages	Diseases predicted using the model
**Random forest** (Number of studies: 58)	-Ability to handle datasets with a large number of predictor variables	-Ignores the spatial locations of the observations so any spatial autocorrelation in the data not accounted for by the covariates.	Alzheimer's disease [28][89] [90] [91] [92] [94] [98] [99] [101]<br>Anxiety [93]<br>Any cardiovascular disorder [29][55] [59] [60] [65]<br>Any mental disorder [100]<br>Asthma [113] [116]<br>Atherosclerosis [64]<br>Atrial fibrillation [53] [124]<br>Chronic obstructive pulmonary disease [118]<br>Chronic kidney disease [87]<br>Colorectal cancer [37] [107] [131] [128] [130]<br>Diabetes mellitus [40] [69] [74] [79]<br>Diabetic polyneuropathy [78] [80]<br>Epilepsy [111]<br>Familial hypercholesterolaemia [31] [39] [82]<br>Fibromyalgia [104]<br>Gestational diabetes [81]<br>Heart Failure [49] [57] [58]<br>Hypertension [51] [62]<br>Inflammatory bowel disease [67]<br>Major depressive disorder [41]<br>Myocardial infarction [43]<br>Obesity [73]<br>Pancreatic cancer [108]<br>Post stroke spasticity [110]<br>Post traumatic stress disorder [96]<br>Preeclampsia [36]<br>Primary Aldosteronism[70]<br>Progressive supranuclear palsy [112]<br>Rheumatoid arthritis [35]<br>Stroke [48]<br>Suicidality [85]
**Support Vector Machine (SVM)**	-Ability to provide non-linear solutions	-Require knowledge about the kernel employed to be able to achieve good performance	Alzheimer's disease [30] [89] [94] [98] [101]<br>Any cardiovascular disorder [52] [55]<br>Any mental disorder [100]

(Number of studies: 30)			Atrial fibrillation [53] [124]<br>Chronic obstructive pulmonary disease [132]<br>Diabetes mellitus [72] [79]<br>Diabetic foot [45]<br>Diabetic polyneuropathy [77] [78] [80]<br>Epilepsy [111]<br>Heart failure [50] [57] [58] [66]<br>Hypertension [51] [56]<br>Influenza [46]<br>Major depressive disorder [41]<br>Post-partum depression [33]<br>Post-traumatic stress disorder [96] [103]<br>Preeclampsia [36]
**Boosting models**<br>(Number of studies: 28)	-Ability to handle categorical features<br>-Few parameters to tune<br>-Good performance in datasets with large number of features	-Interpretability of ensemble can be questioned	Alzheimer's disease [98]<br>Anxiety [93]<br>Any cardiovascular disorder [55] [65] [60]<br>Any mental disorder [100]<br>Asthma [115] [116]<br>Atherosclerosis [64]<br>Chronic obstructive pulmonary disease [118] [120]<br>Colorectal cancer [37] [131] [128]<br>Diabetes mellitus [74] [40]<br>Diabetic polyneuropathy [80]<br>Familial hypercholesterolemia [31]<br>Heart failure [57] [66]<br>Hypertension [51] [56]<br>Lung cancer [109]<br>Non-tuberculous mycobacterial lung disease [114]<br>Obesity [73]<br>Pancreatic cancer [108]<br>Post-partum depression [33]<br>Preeclampsia [36]
**Decision tree techniques**<br>(Number of studies: 25)	-Ability to provide non-linear solutions	-Interpretability of ensemble can be questioned	Alzheimer's disease [98] [101]<br>Ankylosing spondylitis [42] [106]<br>Any cardiovascular disorder [55]<br>Any mental disorder [100]<br>Chronic kidney disease [86]<br>Colorectal cancer [107]<br>COVID-19 [117]

			Diabetes mellitus [40] [68] [75]<br>Diabetic polyneuropathy [77] [78] [80]<br>Heart failure [57]<br>Hypertension[51] [56]<br>Lung cancer [109]<br>Obesity [71]<br>Post-partum depression [33]<br>Preeclampsia [36]<br>Rheumatoid arthritis [35] [42]<br>Stroke [123]
**Naïve Bayes**<br>(Number of studies: 8)	-Good performance in small datasets if conditional independent assumption holds	-Assumption of independence between features	Alzheimer's disease [89] [91] [94] [98]<br>Any cardiovascular disease [63]<br>Epilepsy [111]<br>Major depressive disorder [41]<br>Obesity [71]
**k-nearest neighbors**<br>(Number of studies: 8)	-Intuitive algorithm	-Number of neighbors must be defined by user<br>-High relative computational complexity	Alzheimer's disease [98]<br>Any cardiovascular disease [52] [55]<br>Heart failure [50] [58]<br>Hypertension [51]<br>Diabetes mellitus [32] [72]
**Least absolute shrinkage and selection operator (LASSO)**<br>(Number of studies: 15)	-Good performance with small datasets	-Data assumptions are needed to be complied<br>-Can only provide linear solutions	Anxiety [93]<br>Any cardiovascular disease [29] [60]<br>Atrial fibrillation [53] [124]<br>Back pain [105]<br>Diabetes mellitus [69] [75]<br>Diabetic nephropathy [76]<br>Major depressive disorder [95]<br>Obesity [73]<br>Pancreatic cancer [108]<br>Stroke [123]<br>Suicidality [84]<br>Systematic lupus erythematous [34]
**Artificial Neural Network (ANN)**<br>(Number of studies: 35)	-State-of-the-art results<br>-Direct complex image processing	-Many parameters to fine-tune<br>-Large number of samples are required to achieve good performance	Alzheimer's disease [28] [89] [92] [97] [101] [102]<br>Any cardiovascular disorder [55] [65]<br>Any mental disorder [100]<br>Asthma [116]<br>Atrial fibrillation [53] [124]<br>Chronic kidney disease [86]

			Chronic obstructive pulmonary disease [120]<br>Diabetes mellitus [47]<br>Diabetic polyneuropathy [78]<br>Diabetic retinopathy [44] [38] [125][126] [127]<br>Familial hypercholesterolemia [31]<br>Gastroesophageal reflux [26]<br>Heart failure [49] [50] [57]<br>Hypertension [54] [61]<br>Influenza [119]<br>Pancreatic cancer [108]<br>Post-traumatic stress disorder [96] [103]<br>Preeclampsia [36]<br>Psychosis [122]<br>Suicidality [83]
