# Supplementary material for: A systematic review of clinical health conditions predicted by machine learning diagnostic and prognostic models trained or validated using real-world primary health care data
Source: PLoS One. 2023 Sep 8;18(9):e0274276. doi: 10.1371/journal.pone.0274276 (PMC10491005; doi:10.1371/journal.pone.0274276)
Supplement: S1 File — (PDF) [file pone.0274276.s003.pdf]

Search strategy

PICOTS:

- **Population**: patients with any health condition within primary health care.
- **Index test**: Machine learning prediction models.
- **Comparator**: usual care, any other non-machine learning model, or none.
- **Outcome**: specific primary outcome of each health condition.
- **Timing**: before or at diagnosis point and short- or long-term follow up points.
- **Setting**: ambulatory settings worldwide.

Search string:

Literature search using only population and index test search strings. Primary outcomes of health conditions were not used as search terms for the wide scope of the review. Search string were as follows:

Population	Index test
Primary health care [MeSH Term]<br>Primary healthcare / Primary care<br>General practice [MeSH Term]<br>Family practice / Family medicine<br>Ambulatory care / Ambulatory setting<br>Nursing	Machine Learning [MeSH term]<br>Artificial intelligence [MeSH Term]<br>Deep Learning [MeSH Term]

Database search:

Applied filters: 1990-2022, primary studies and reviews, and all fields/full-text search. Search was conducted on **05.08.2021 and updated on 04.01.2022.**

**Web of Science (include 8 databases)**	
TS=("machine learning" OR "artificial intelligence" OR "deep learning") Databases= WOS, BCI, BIOSIS, CCC, KJD, MEDLINE, RSCI, SCIELO Timespan=1990-2022, Search language=Auto	501,34
TS=("primary health care" OR "primary healthcare" OR "primary care" OR "general pract*" OR "family pract*" OR "ambulatory setting*" OR nurs* OR "ambulatory care" OR "family medicine")<br>Databases= WOS, BCI, BIOSIS, CCC, KJD, MEDLINE, RSCI, SCIELO Timespan=1990-2022<br>Search language=Auto	1,077,792
#1 AND #2	2,141
**PubMed**	
("machine learning" OR "artificial intelligence" OR "deep learning") Filters applied: Clinical Trial, Observational Study, Randomized Controlled Trial, Review, Systematic Review, Validation Study	10,974
("primary health care" OR "primary healthcare" OR "primary care" OR "general pract*" OR "family pract*" OR "ambulatory setting*" OR nurs* OR "ambulatory care" OR "family medicine")<br>Filters applied: Clinical Trial, Observational Study, Randomized Controlled Trial, Review, Systematic Review, Validation Study	194,847
#1 AND #2	340
**Cochrane Library**	
("machine learning" OR "artificial intelligence" OR "deep learning") with Cochrane Library publication date Between Jan 1990 and Jan 2022, in Cochrane Reviews, Trials (Word variations have been searched)	2,292

("primary health care" OR "primary healthcare" OR "primary care" OR "general pract*" OR "family pract*" OR "ambulatory setting*" OR nurs* OR "ambulatory care" OR "family medicine") with Cochrane Library publication date Between Jan 1990 and Jan 2022, in Cochrane Reviews, Trials (Word variations have been searched)	86,323
#1 AND #2	138
**Elsevier-Embase**	
('machine learning'/exp OR 'machine learning' OR 'artificial intelligence'/exp OR 'artificial intelligence' OR 'deep learning'/exp OR 'deep learning') AND [1990-2022]/py	293,714
('primary health care' OR 'primary healthcare' OR 'primary care' OR 'general pract*' OR 'family pract*' OR 'ambulatory setting*' OR nurs* OR 'ambulatory care' OR 'family medicine') AND [1990-2022]/py	1,465,194
#1 AND #2	4,973
**Elsevier-Scopus**	
ALL ({machine learning} OR {artificial intelligence} OR {deep learning}) AND PUBYEAR > 1989 AND (LIMIT-TO (SUBJAREA , "COMP") OR LIMIT-TO (SUBJAREA , "MEDI") OR LIMIT-TO (SUBJAREA , "HEAL") OR LIMIT-TO (SUBJAREA , "NURS")) AND (LIMIT-TO (DOCTYPE , "ar") OR LIMIT-TO (DOCTYPE , "re"))	845,187
ALL ({primary health care} OR {primary healthcare} OR {primary care} OR {general pract*} OR {family pract*} OR {ambulatory setting*} OR {nurs*} OR {ambulatory care} OR {family	776,692

medicine}) AND PUBYEAR > 1989 AND (LIMIT-TO (SUBJAREA , "MEDI") OR LIMIT-TO (SUBJAREA , "NURS") OR LIMIT-TO (SUBJAREA , "HEAL") OR LIMIT-TO (SUBJAREA , "COMP")) AND (LIMIT-TO (DOCTYPE , "ar") OR LIMIT-TO (DOCTYPE , "re"))	
#1 AND #2	9,975
**Elsevier-ScienceDirect**	
("machine learning" OR "artificial intelligence" OR "deep learning") 1990-2021, reviews, articles, medicine and dentistry, computer science	94,611
("primary health care" OR "primary healthcare" OR "primary care" OR "general practice" OR "family practice" OR "ambulatory setting" OR nurs OR "ambulatory care" OR "family medicine") 1990-2022, reviews, articles, medicine and dentistry, Nursing and health profession	168,917
#1 AND #2	2,358
**BioRxiv and MedRxiv**	
"machine learning" and posted between "01 Jan, 1990 and 04 Jan, 2022"	16,363
"primary health care" and posted between "01 Jan, 1990 and 04 Jan, 2022"	633
"general practice" and posted between "01 Jan, 1990 and 04 Jan, 2022"	933
#1 AND #2OR#3, subjects: all	10,333
#1 AND #2OR#3, subjects: Primary care research, nursing, bioinformatics	1,259
**ACM Digital Library**	
[All: "machine learning"] OR [All: "artificial intelligence"] OR [All: "deep learning"] AND [Publication Date: (01/01/1990 TO 01/04/2022)]	19,970

[All: "primary health care"] OR [All: "primary healthcare"] OR [All: "primary care"] OR [All: "general pract*"] OR [All: "family pract*"] OR [All: "ambulatory setting*"] OR [All: nurs*] OR [All: "ambulatory care"] OR [All: "family medicine"] AND [Publication Date: (01/01/1990 TO 01/04/2022)]	5,589
1# AND #2	1,701
**IEEE Xplore Digital Library**	
("All Metadata":"machine learning" OR "All Metadata":"artificial intelligence" OR "All Metadata":"deep learning") Filters Applied: Journals, 1990 - 2022	54,451
("All Metadata":"primary health care" OR "All Metadata":"primary healthcare" OR "All Metadata":"primary care" OR "All Metadata":"general pract*" OR "All Metadata":"family pract*" OR "All Metadata":"ambulatory setting*" OR "All Metadata":nurs* OR "All Metadata":"ambulatory care" OR "All Metadata":"family medicine")<br>Filters Applied: Journals, 1990 - 2022	601
1# AND #2	160
Total retrieved studies	23,045
