# Supplementary material for: A systematic review of clinical health conditions predicted by machine learning diagnostic and prognostic models trained or validated using real-world primary health care data
Source: PLoS One. 2023 Sep 8;18(9):e0274276. doi: 10.1371/journal.pone.0274276 (PMC10491005; doi:10.1371/journal.pone.0274276)
Supplement: S1 Panel — (PDF) [file pone.0274276.s005.pdf]

S1 Panel. Quantitative summary of the included studies.

➢ **Total articles included:**<br>106	➢ **Total number of participants:**<br>24.2 million
➢ **Publications by year:**<br>• 2002, 2008, 2014: one article each<br>• 2010, 2013: three articles each<br>• 2015: two articles<br>• 2016: five articles<br>• 2017: seven articles<br>• 2018: ten articles<br>• 2019: 22 articles<br>• 2020: 25 articles<br>• 2021: 25 articles<br>• 2022: (up to Jan 4’ one article)	➢ **Countries under study: time reported**<br>• United States of America: 41<br>• United Kingdom: 31<br>• Netherlands: 11<br>• Canada: 9<br>• Spain: 6<br>• Germany: 4<br>• Sweden, China, Australia: 3 each<br>• Russia, Denmark, Indonesia, Israel, Kuwait: 2 each<br>• Croatia, Taiwan, Italy, Slovenia, Ukraine: 1 each
➢ **Study design:**<br>• Pros. cohort: 8 articles<br>• Retro. cohort: 63 articles<br>• Retro. case control: 29 articles<br>• Retro. nested case control: three articles<br>• Cross sectional: three articles	➢ **Models’ Purpose: 107**<br>• Inc. diagnostic: 62<br>• Prev. diagnostic: 20<br>• Prognostic: 25
➢ **Type of modelling studies:**<br>• Development without external validation: 81<br>• Development with external validation: 13<br>• External validation: 12	➢ **Primary care data sources: time used for models’ development and/or validation: 126**<br>• Exclusive PHC data: 92<br>• Linked PHC data: 34
➢ **Machine Learning models used: 207**<br>• Supervised ML: 172<br>• Reinforcement ML: 35<br><br>Natural language processing NLP: 5 (a preparatory step)	
➢ **Health conditions predicted (according to ICD-10 - 2019) (n= 42 across 106 studies)**<br><br>**1. Endocrine, metabolic, nutritional diseases: 27**<br>• Diabetes mellitus: 10<br>• Diabetic retinopathy: 5<br>• Diabetic polyneuropathy: 3<br>• Familial hypercholesterolemia: 3	

- Children obesity: 2
- Primary Aldosteronism: 1
- Diabetic foot: 1
- Diabetic nephropathy: 1
- Gestational Diabetes: 1

2. **Circulatory: 23**
- Any cardiovascular disease: 7
- Heart failure: 5
- Hypertension: 5
- Atrial fibrillation: 2
- Stroke: 2
- Atherosclerosis: 1
- Myocardial infarction: 1

3. **Mental and behavioral disorders: 21**
- Alzheimer’s disease: 12
- Major depressive disorder: 3
- Post-partum depression: 1
- Psychosis: 1
- Post-traumatic stress disorder: 2
- Anxiety: 1
- Any mental disorder: 1

4. **Respiratory system diseases: 10**
- Chronic obstructive pulmonary disease: 3
- Asthma: 2
- Influenza: 2
- Asthma/COPD overlap: 1
- COVID-19 : 1
- Nontuberculous mycobacterial lung: 1

5. **Neoplasms: 8**
- Colorectal cancer: 6
- Lung cancer: 1
- Pancreatic cancer: 1

6. **Musculoskeletal and connective tissue diseases: 6**
- Rheumatoid arthritis: 2
- Ankylosing spondylitis: 1
- Back pain: 1
- Fibromyalgia: 1
- Systemic lupus erythematosus: 1

7. **Nervous: 3**
- Post stroke spasticity: 1
- Epilepsy: 1

- Progressive Supranuclear Palsy: **1**

8. External cause of mortality: 3

- Suicidality: 3

9. Digestive: 2

- Gastroesophageal reflux: 1
- Inflammatory bowel diseases: 1

10. Genitourinary: 2

- Chronic kidney disease: 2

11. Pregnancy, childbirth, puerperium: 1

- Preeclampsia: 1
