# Supplementary material for: A systematic review of clinical health conditions predicted by machine learning diagnostic and prognostic models trained or validated using real-world primary health care data
Source: PLoS One. 2023 Sep 8;18(9):e0274276. doi: 10.1371/journal.pone.0274276 (PMC10491005; doi:10.1371/journal.pone.0274276)
Supplement: Attachment — Submitted filename: PONE-D-22-23382.docx [file pone.0274276.s006.docx]

This work presents a systematic review of the application of ML in the context of primary health care. Literature published between January 1990 to January 2022 is considered.

1. No insights related to algorithms applied. Any observation related to the most commonly used algorithms and the best performing ones?
2. What kind of data is usually used? Textual? Images? What kind of features?
3. What ate the current challenges in applying ML to primary health care data?
4. What are the most challenging diseases for ML prediction?
5. What kind of evaluation approaches are used?
6. Has any of the reviewed work been deployed in a real world application?
7. Quality of figures and tables need to be improved.
8. Need to discuss implications of the results.
