# Supplementary material for: A systematic review of clinical health conditions predicted by machine learning diagnostic and prognostic models trained or validated using real-world primary health care data
Source: PLoS One. 2023 Sep 8;18(9):e0274276. doi: 10.1371/journal.pone.0274276 (PMC10491005; doi:10.1371/journal.pone.0274276)
Supplement: Attachment — Submitted filename: Response to Reviewers-R2.docx [file pone.0274276.s008.docx]

**Revision 2: Response to Reviewers' Comments**

Our responses are written in blue.

**Response to Reviewer #2:**

We would like to thank you for your valuable comments that helped us improve our manuscript. We are very eager to properly address all your comments. Despite that, it seems that we did not address some of your comments. We apologize for this.

We now addressed all comments in detail, restructured parts of the discussion and added a new table (S2 Table) to the supplement addressing the characteristics of all ML predictive models included in the manuscript (see below).

1. No insights related to algorithms applied. Any observation related to the most commonly used algorithms and the best performing ones?

- We addressed this point in our manuscript through presenting the advantages and disadvantages of the included models and related that to our results across the manuscript:
  - We referred to it in the introduction section in page 3, lines 60-61 as follows “The variety of ML prediction models’ characteristics provide broader opportunities to support the healthcare practice.”
  - We presented more information regarding the advantages and disadvantages of the models included in a new supplementary Table S2. We referred to it in our results section page 10, lines 200-201 as follows: “Supplementary table (S2 Table) presents advantages and disadvantages of these models in addition to further descriptive information of the included studies.”
  - In the discussion section, we discussed the most commonly used models in page 20, line 323-325 as follows: “In 106 observational studies, we identified 42 health conditions targeted by 207 ML prediction models, of which 38% were random forest and SVM. The included models used PHC data documented over the past 40 years for a total of 22.7 million patients.”. Please also see answer to question 2 below.
  - Furthermore, we related our results to the advantages of these models and the potential benefit of it within the primary healthcare and referred to it in page 22, lines 360-369 as follows: “Models that are known for their high prediction accuracy, such as random forest and SVM might support better health outcomes when developed using high quality health data [140]. Additionally, the variety of the ML prediction models characteristics provide opportunities to improve healthcare practice. Using large data documented as electronic health records, random forest models and ensemble models such as boosting models have the ability to handle large datasets with numerous predictors variables [141]. Artificial neural network can also perform complex images processing that can boost the primary health care services [141]. Furthermore, SVM and decision tree models can provide nonlinear solutions, thus will support our understanding of complex and dynamic diseases for earlier health conditions prediction [142].”

2. What kind of data is usually used? Textual? Images? What kind of features?

We added a paragraph to highlight the data used in page 10, lines 207-2014, as follows: “The data used to develop the models were called predictors, features, or variables across the included studies. These data were mostly textual. Demographic characteristics and clinical picture of the health conditions were the most frequently found data. Medications, comorbidities, and blood tests performed within primary care unit were reported. Data, such as blood test results and imaging results performed within secondary and tertiary health care were additionally reported in some of the individual studies. Referral documentation and clinical notes taken by health care personnel were also reported. Five studies used the natural language processing (NLP) technique to handle free text clinical notes [43,45–48].”

3. What are the current challenges in applying ML to primary health care data?

- We highlighted some of the current challenges in applying ML to PHC data in the discussion section and presented it in context with our results in the subsection “Results in perspective” in two places:
  - in page 21, lines 344-352 as follows: “The coding system used in health records does not universally follow the same criteria for all diseases, posing challenges for the consistency of models’ performance [137]. Moreover, the lack of globally standardized definitions and terminology of diseases and the wide variability of the services provided across different health systems further limit the effectiveness of the models [137]. For example, uncoded free-text clinical notes as well as using ‘race’ and ‘ethnicity’ or ‘suicide’ and ‘suicide attempts’ to be documented as a single input can affect the predictive power of the models [138]. Other drawbacks reported include underrepresentation of healthy persons, retrospective temporal dimension of predictors, and the absence of confirmatory diagnostic services in PHC pose significant limitations [140, 14 1].”
  - in page 22, lines 378-386, as follows “Health care professionals are fundamental to the process of implementing and integrating ML prediction models in their healthcare practice. Despite that, our review did not report outcomes related to healthcare professionals. Significant variability of opinions on the utilization of ML in PHC among primary health care providers hinder its acceptance. Furthermore, the black-box nature of ML prediction models precludes the clinical interpretability of models’ outcomes. Additional workload and training are needed to implement such technology in the routine practice. Trust, data protection, and ethical and clinical responsibility legislation are further intractable issues that represent major obstacles toward ML prediction models implementation [5].”
  - In page 24, lines 410-413, as follows “Despite the additional burden, it is beneficial also to continuously assess the potential significance of models, such as improved health outcomes, reduced medical errors, increased professional effectiveness and productivity, and enhanced patients’ quality of life [147].”

4. What are the most challenging diseases for ML prediction?

- We addressed this point in page 22, lines “370-377” as follows: “Nature of diseases append further challenges. The most challenging diseases for ML prediction are multifaceted long-term health conditions, such as DM, that are influenced by combination of genetic, environmental, and lifestyle factors. The complex health conditions further tangle the models, making it harder to identify accurate predictive patterns. Furthermore, the subjective nature of symptoms, especially symptoms related to mental health disorders, pose additive challenges toward ML models accuracy. Rare diseases, if documented, often suffer from limited data availability, leading to difficulty to train ML models effectively [143].”

5. What kind of evaluation approaches are used?

- We addressed this point in page 10, lines 201-206 as follows: “The most frequently reported evaluation approach of models’ performance was the area under the receiver operating characteristic curve (AUROC), which was reported as “good” to “moderate” models performance in 62 studies. One study reported the performance measures using decision analysis curve [32]. Other evaluation approaches were reported across the included studies, such as calculating sensitivity, specificity, predictive values, and accuracy.”

6. Has any of the reviewed work been deployed in a real world application?

- We referred to this point in page 9, lines 190-192 “Despite all studies included used real-world patients' data to develop and/or validate the ML models, four studies reported applying the models develop in real-world primary health care settings [127,128, 130, 131].”

7. Quality of figures and tables need to be improved.

- We improved the quality of all our tables and figure by proper reformatting for font, spacing, and colours.

8. Need to discuss implications of the results.

- The discussion section was re-written and reorganized to highlight the implication of results. We added a subsection in pages 23-24, lines 393-422 as follows “Implication of results and recommendation for future contributions: This review provided a comprehensive outline of ML prediction models in PHC and raises important considerations for future research and implementation of this technology in PHC settings. Interdisciplinary collaboration among health care workers, developers of ML models, and creators of digital documentation systems is required. This is especially important given the increasing popularity of digitally connected health systems [5]. It is recommended to augment the participation of health professionals through the development process of the PHC predictive models to critically evaluate, assess, adopt, and challenge the validation of the models within practices. This collaboration may assist ML engineers to recognize unintended negative implications of their algorithms, such as accidentally fitting of confounders, and unintended discriminatory bias, among others, for better health outcomes [146]. Health care systems need to provide comprehensive population health data repositories as an enabler for medical analyses [137]. Well-designed and -documented repositories which provide representative health data for the healthy and diseased populations are needed [137, 140]. These high quality data repositories might provide future modelling studies with data that match the studies’ clinical research questions for more accurate prediction. Further ML prediction studies are needed to target more health conditions using PHC data. Despite being challenging, it is beneficial to early assess the potential significance of models, such as improved health outcomes, reduced medical errors, increased professional effectiveness and productivity, and enhanced patients’ quality of life [147]. It is recommended to follow reporting guidelines for producing valid and reproducible ML modelling studies. Developing robust frameworks to enable the adoption and integration of ML models in the routine practice is also essential for effective transition from conventional health care systems to digital health [148,149]. Sophisticated technical infrastructure and strong academic and governmental support are essential for promoting and supporting long-term and broad-reaching PHC ML-based services [138,150]. However, balanced arguments [151,152] regarding the potential benefits and limitations of ML models support better health care without overestimating or hampering the use of such technology. It is also suggested to integrate the basic understanding of ML concepts and techniques in education programs for health science and medical students.”
